# Adenovirus 14p1 Immunopathogenesis during Lung Infection in the Syrian Hamster

**DOI:** 10.3390/v12060595

**Published:** 2020-05-30

**Authors:** Jay R. Radke, Hunter J. Covert, Fredrick Bauer, Vijayalakshmi Ananthanarayanan, James L. Cook

**Affiliations:** 1 Research Section, Edward Hines, Jr. VA Hospital, Hines, IL 60141, USA; jacook@luc.edu; 2 Department of Microbiology and Immunology, Loyola University of Chicago—Stritch School of Medicine, Maywood, IL 60153, USA; 3 Infectious Diseases and Immunology Research Institute, Loyola University Chicago—Stritch School of Medicine, Maywood, IL 60153, USA; 4 Division of Infectious Diseases, Loyola University Chicago—Stritch School of Medicine, Maywood, IL 60153, USA; 5 Research Section, Boise VA Hospital, Boise, ID 83702, USA; hunter.covert33@gmail.com (H.J.C.); Frederick.Bauer@va.gov (F.B.); 6 Idaho Veterans Research and Education Foundation, Boise, ID 83702, USA; 7 Department of Pathology, Loyola University Chicago—Stritch School of Medicine, Maywood, IL 60153, USA; VANANTH@lumc.edu

**Keywords:** adenovirus, acute lung injury, Syrian hamster, acute respiratory distress syndrome

## Abstract

Adenovirus (Ad) infections are usually mild and self-limited, with minimal inflammatory responses. During worldwide outbreaks, Ad14p1, an emerging Ad14 variant, has caused severe pulmonary disease, including acute respiratory distress syndrome (ARDS). This increased pathogenicity of Ad14p1 is not completely understood. In initial studies, we observed that infection of Syrian hamsters with Ad14p1 can cause a patchy bronchopneumonia, with an increased intensity of inflammation, compared to wild type Ad14 infection. The current study compared the dynamics of the immunopathogenesis of Ad14 and Ad14p1 infection of hamster lungs through the first two weeks after infection. Little difference was seen in infection-induced inflammation at day 1. Beginning at day 3, Ad14p1-infected hamsters showed marked inflammation that continued through to day 7. The inflammation began to resolve by day 10 but was still detectable at day 14. In contrast, Ad14-infected hamsters showed little inflammation during the 14-day period of observation. Inflammatory cell type analysis revealed that, at day 1, hamsters infected with either virus had predominantly neutrophil infiltration that began to resolve by day 3. However, at day 5, Ad14p1-infected hamsters had a second wave of neutrophil infiltration that was accompanied by edema which persisted to a variable extent through to day 10. These differences were not explained by an increased Ad14p1 replication rate, compared with Ad14 in vitro, but there was prolonged persistence of Ad14p1 in hamster lungs. There were differences in lung tissue cytokine and chemokine responses to Ad14p1 vs. Ad14 infection that might account for the increased leukocyte infiltrates in Ad14p1-infected hamsters. This animal model characterization provides the basis for future translational studies of the viral genetic mechanisms that control the increased immunopathogenesis of the emergent, Ad14p1 strain.

## 1. Introduction

Adenovirus (Ad) infection usually induces mild, self-limited infections in immunocompetent human hosts. In immunocompromised individuals, Ad infection can be severe and often fatal. However, outbreaks of emergent strains of Ad have resulted in severe and fatal infections in otherwise healthy individuals, as well as those with underlying lung disease and immunologic compromise.

Ad14 (deWit) was first isolated in the Netherlands in 1955 following an acute respiratory disease (ARD) outbreak in the military [1]. After cancelation of the US military adenovirus vaccination program, there was a spike in Ad4 and Ad7 infections, the predominant Ad strains observed in the US military [2]. At the same time, Ad14 appeared for the first time in US military populations [2]. Sequencing revealed that these emerging Ad14 strains were 100% identical in the coding regions but were distinct from the prototype Ad14 deWit strain (isolates share 99.6% identity with prototype). The emergent strain was classified as Ad14p1 [3,4,5,6]. Currently, the Ad14p1 strain is circulating worldwide and has been identified as the causative agent in several acute outbreaks associated with an increased incidence of acute respiratory distress syndrome (ARDS). These outbreaks have had approximately a 10% case fatality rate, with deaths observed in both immunocompromised and immunocompetent patients [7,8,9,10,11]. The increased severity of infection of immunocompetent patients with Ad14p1 is of great concern. There appear to be no consistent risk factors predicting the outcome of Ad14p1 infection; however, older age, a history of smoking and underlying conditions have been observed in some case series [8,12]. Based on the epidemiology data, it has been suggested that there are other patient and environmental factors that determine the outcome of infection [13].

The best described clinical case of Ad14p1 was that of a 74 year-old woman in Canada [14]. She presented to the hospital after three days of influenza like-illness, and a chest X-ray revealed bilateral alveolar infiltrates. Other than a history of smoking, she had no remarkable medical history. She was admitted to the ICU and progressed to ARDS and died six days later. Nasopharyngeal and tracheal aspirations were negative for influenza by PCR. The patient also tested negative for pneumococcus, *Legionella* spp. and hantavirus. Analysis of respiratory specimens was negative for bacteria but showed an abundance of leukocytes. Virus culture showed cytopathic effect (CPE) at six days, and sequence analysis confirmed Ad14p1 infection. Postmortem pathology revealed dark purple lungs with marked consolidation and patchy hemorrhagic foci. Histopathology showed diffuse alveolar damage (DAD), fibrinohemorrhagic changes, areas of necrosis and cellular debris. Immunohistochemistry revealed few Ad-positive epithelial cells with cytoplasmic granular and nuclear staining.

Initial in vitro studies of the comparative viral fitness of Ad14p1 vs. Ad14 demonstrated little difference in the infectivity and growth rate in established human cell lines [15]. Using differentiated primary human bronchial epithelial cells, Lam et al. showed that Ad14p1 is released predominantly from the apical rather than basolateral cell surface [16]. Furthermore, Ad14p1 infection of the bronchial epithelial cells resulted in increased expression of IL-6 and the chemoattractant chemokines CXCL10 (IP-10) and CXCL11 compared to infection with Ad5 [16]. They speculated that the apical release of Ad14p1 might facilitate infection of the lower respiratory tract and that increased expression of IL-6 and IP-10 and CXCL11 might contribute to the increased host inflammatory response.

We have reported that cells dying as a result of adenovirus serotype 5 (Ad5) infection repress the host inflammatory response through a mechanism that requires the expression of the adenoviral gene product, E1B 19K [17]. We hypothesized that emergent Ad strains that induce ARDS might either lack sufficient expression of 19K or express a mutated 19K resulting in an enhanced host pro-inflammatory response to infection. To test this hypothesis, we did the same studies with wild type Ad14 and the emergent Ad14p1 strain. Cells infected with Ad14p1 expressed only 20% of the amount of E1B 20K (the Ad14 equivalent of Ad5 E1B 19K) compared to Ad14-infected cells [18]. The resulting Ad14p1-infected dying cells failed to repress pro-inflammatory cytokine production from primary alveolar macrophages (AM). Initial in vivo studies revealed that intratracheal infection of Syrian hamsters with Ad14p1 resulted in pronounced bilateral pneumonias at day 7 post infection, whereas hamsters infected with Ad14 showed only minor inflammation. These results suggested that Syrian hamsters might be useful as a small animal model to characterize the immunopathogenesis of Ad14p1-induced lower respiratory tract infection, as a basis for translational studies of the viral genetic determinants that control the intensity and quality of the host inflammatory response [18]. The objective of the current study was to characterize this model of comparative Ad14 vs. Ad14p1 infection and the evolving host innate immune response and related lung immunopathology.

In this study we show that infection with Ad14p1 results in an ALI/ARDS like response in the Syrian hamster. Histological studies showed that Ad14p1 induces a patchy bronchopneumonia that is not observed in Ad14-infected hamsters, as well as prolonged Ad14p1 persistence and intense inflammation in the lung. The inflamed regions of the lungs of animals infected with both viruses contained macrophages, neutrophils and lymphocytes; however, the inflammation was more intense in animals infected with Ad14p1 than those infected with Ad14. Both Ad14 and Ad14p1 induced a rapid neutrophil infiltration into the airways at day 1 post infection (p.i.) that began to resolve by day 3 p.i., but Ad14p1 induced a second wave of neutrophil infiltration at day 5 p.i. that persisted through to day 7 p.i. These differences in the inflammatory responses to infection were not caused by a less effective viral infection by the two viruses, since initial lung tissue viral loads were comparable. Infection with Ad14p1 resulted in increased expression of pro-inflammatory cytokines and chemokines compared to Ad14. The data presented here indicate that the Syrian hamster can be used as a model to study the viral genetic control of the infection-induced, early, ALI in response to Ad14p1 infection that might result in an ARDS-like outcome.

## 2. Materials and Methods

### 2.1. Viruses

Prototype adenovirus type 14 deWit (Ad14; VR-15; herein referred to as Ad14) was obtained from ATCC. Ad14p1 was obtained from Kevin Russell (US Naval Health Research Center, San Diego, CA, USA) and Adriana Kajon (Lovelace Respiratory Research Institute, Albuquerque, NM, USA). Virus stocks were grown in A549 cells and purified by CsCl banding. Adenovirus titers were determined by qPCR using a reference stock of virus that was plaque tittered in triplicate for the standard curve and expressed as genomes/mL [18]. Both Ad14 and Ad14p1 had particle to infectivity ratios of 10:1.

### 2.2. Animals

Four to six-week-old female *Mesocricetus auratus* (Syrian hamster) were obtained from Envigo (Indianapolis, IN, USA) and had access to food and water ad libitum. Hamsters were allowed to acclimate for 72 h after shipping, before experimental use.

### 2.3. Ethics Statement

Studies were conducted in strict accordance with recommendations of the Guide for the Care and Use of Laboratory Animals of the National Institutes of Health and approved by both the Hines, VA, USA (H14-001; 7 January 2014 to 28 May 2017) and Boise, VA, USA (RDIS#0001; 25 September 2017) IACUC committees. Hamsters were anesthetized with 2.5% isoflurane delivered in a stream of oxygen by a controlled precision vaporizer. Hamsters were euthanized using CO_2_ (Hines, VA, USA) or sedated with isoflurane followed by cervical dislocation for bronchoalveolar lavage (Boise, VA, USA).

### 2.4. Viral Infection of Animals

Hamsters were infected as previously described [18]. Briefly, hamsters were sedated with isoflurane to both calm them and induce slower, deeper respiration. Ad14 or Ad14p1 at 5 × 10^9^ genomes were instilled in hamsters in the upright but slightly recumbent position via intratracheal aspiration during panting respiration. Hamsters were held in this position for 30 breaths before being placed in their cages for anesthesia recovery.

### 2.5. Histopathology of Lungs

At the given times post infection (p.i.), hamsters were euthanized by CO2 asphyxiation. Lungs were removed and inflated with 10% neutral buffered formalin, then placed in tissue cassettes and fixed in formalin for at least 24 h before being embedded in paraffin. Sections (3–5 µm) were prepared for both histopathology and immunohistochemistry. Sections for pathology were stained with hematoxylin and eosin (H&E). Clinical pathologists with experience in microbiology and infectious diseases blindly reviewed the slides. A-component and B-component inflammation were scored according the 2007 revision for grading lung allograft rejection [19]. Epithelial cell damage and hyperplasia in the proximal and distal bronchioles were defined as: necrotic cells with cytoplasmic condensation, karyorrhexis, or pyknosis; reactive cells with cystic spaces; and hyperplasia with stratification and loss of mitotic figures, loss of cilia. These were graded from 0–4 with absent-G0, focal-G1, non-circumferential-G2, complete circumferential-G3, and circumferential with destruction-G4. Alveolar inflammation was scored 0–4 based on severity with G4 being severe with hyaline membranes. Bronchial inflammation was scored 0–4 with absent-G0, patchy-G1, >50%-G2, circumferential-G3, circumferential and rim of lymphocytes-G4. Perivascular inflammation was scored 0–4 with absent-G0, patchy-G1, >50%-G2, circumferential and invasion of vascular wall-G3, and destruction of vascular wall-G4. Interstitial inflammation was scored from 0–4 with absent-G0, 1–2 cell-thick interstitial inflammation-G1, expansion of the interstitium with mononuclear infiltrate-G2, expansion of the interstitium with consolidation-G3, expansion of the interstitium with consolidation and necrosis-G4. Type II pneumocyte hyperplasia was scored on severity from 0–4 with G4 involving pneumocyte necrosis. Necrosis and sloughing was defined as cells and debris within any respiratory (non-alveolar) airway and was scored as either absent or present. Presence of adenovirus inclusions was also noted. Differential cell counts in inflamed areas were performed on 3 µm sections and were only possible on the peripheral edges of the inflamed areas due the density of cells in the interior.

### 2.6. Immunohistochemistry

Deparaffinization was performed in a series of xylene washes followed by rehydration in graded alcohols. Unmasking was performed with Triology (Cell Marque, Rocklin, CA, USA) in a pressure cooker. Tissue was permeabilized with 0.2% Triton X-100-TBS and incubated for 10 min at RT. Slides were washed with IHC Wash Buffer (Cell Marque) followed by incubation with Peroxidase Block (Cell Marque) for 10 min at RT. Slides were then washed and blocked in 5% hamster serum diluted in the 0.2% Triton X-100-TBS for 1 h at RT. Slides were washed after blocking. Primary antibodies were diluted in blocking solution and incubated with slides for 2 h at RT in the dark. Tissues were incubated with anti-CD68 (1:300; LS-Bio, LS-C343891, Seattle, WA, USA), rabbit polyclonal anti-CD3ε (1:500; Novus, NB600-1441), anti-MPO (0.5 µg; LS-Bio, LS-C388920) or anti-GFP (1:500; Abcam, ab290) as isotype control. Slides were washed with IHC wash buffer and incubated with HiDef Detection HRP Polymer Detection (Cell Marque) as recommended. DAB Substrate kit was used for color development. Tissues were counterstained with Hematoxylin 7211 (Fisher, Waltham, MA USA). Dehydration was done using graded alcohols followed by a final rinse in xylene before mounting. A serial section of lung was stained for Ad-infected cells with a pan-anti-hexon antibody mix (adenovirus 20/11 and 2/6; Cell Marque) [18].

### 2.7. Recovery of Immune Cells from Bronchoalveolar Lavage Fluid

Hamsters were euthanized according to approved protocols. Tracheas were exposed, cannulated in between the two superior cartilage rings with a 16 G hypodermic needle sheathed with tubing and secured with a suture. Lungs were lavaged twice, the first consisting of an instillation of 4 mLs of PBS to inflate the lungs and immediately withdrawn and collected. A second 4 mLs of PBS was instilled and flushed through the lungs three times and then pooled with the first lavage. ACK was used to lyse any red blood cells followed by washing with PBS.

### 2.8. Differential Cell Counts on BALF Cells

Cells from BALF (1 × 10^5^) were aliquoted into 300 µL PBS and gently mixed. Two drops of 22% bovine serum albumin (Immuncor, Norcross, GA, USA) was added to cells that were then gently mixed and spun onto Superfrost Plus charge slides (Fisher) for 10 min at 900 rpm (Shandon Cytospin 2). Slides were allowed to air dry and then stained with Wright Giemsa.

### 2.9. Quantitation of Ad14 Genomes in Hamster Lungs

Slices of lung were weighed, homogenized in HBBS (Qiagen, Germantown, MD, USA) and subjected to three rounds of freeze/thaw cycles. Detection of viral genomes in lung samples was done using qPCR as previously described [18]. Primers used for Ad14 quantitation were 5′-CGGAGCTGCCTGGACATG-3′ and 5′-GCTTIACAGGAATGGGCTTG-3′, which amplifies Ad serogroup B *E1A*. iTaq Universal SYBR Green Supermix (Bio-Rad, Hercules, CA, USA) was used as the qPCR reagent. qPCR was performed on a Light Cycler 480 II (Roche Molecular Systems, Pleasanton, CA, USA). The qPCR protocol was: 95 °C for 3 min (1×)/ 95 °C for 30 s; 55 °C for 1:30 min; 72 °C for 1 min (40×); data collection occurred at the end of the 72 °C amplification step. Following amplification, melt curve analysis (55.0–95.0 °C; 0.5 °C/read; 5 s dwell time) was performed. All qPCR reactions were performed in 96-well optical plates with optical adhesive seals. Viral genomes present in lungs were expressed as genomes/g of lung.

### 2.10. Cytokine Gene Expression

One lobe of the right lung was removed and frozen using RNAlater (Invitrogen, Thermo Fisher Scientific, Waltham, MA, USA). Total RNA was isolated from homogenized lung slices with RNeasy Mini Kit (Qiagen). Superscript IV (Invitrogen) was used to generate first strand cDNA. Primers (Invitrogen) for gene expression were described by Zivcec et al. [20]. PCR reaction consisted of 33 ng of cDNA template, 0.4 µM primers in iTaq Universal SYBR Green Supermix (Bio-Rad). The qPCR was performed on a Light Cycler 480 II (Roche) with the following PCR conditions: 95 °C for 5 min (1×)/ 95 °C for 30 s; 60 °C for 2 min (40×); data collection occurred at the end of the 60 °C step. RPL18 served as the normalization control for each gene from each hamster (ΔCT) [20]. Each normalized gene expression result from infected hamsters was compared with the normalized gene expression from uninfected controls to determine the ΔΔCT. Final values were expressed as fold induction (log2) compared to uninfected hamsters.

### 2.11. Statistical Analysis

Graph Pad Prism 8 was used to perform statistical analysis. A one-way ANOVA was performed followed by post-hoc analysis by the Sidak method to determine significance of differences between two sets of data. For all analyses, *p* < 0.05 was considered to be a significant difference.

## 3. Results

### 3.1. Ad14p1 Induces Severe Inflammation in the Lungs of Syrian Hamsters

**Figure 1:**
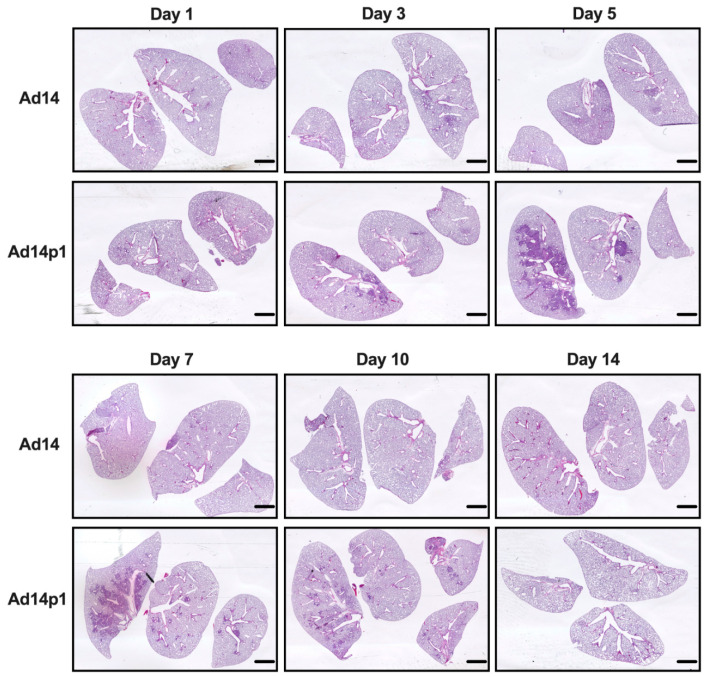
Effects of Ad14 and Ad14p1 on Syrian hamster lung pathology. H&E staining of Syrian hamster lungs post infection with 5 × 10^9^ genomes/animal via intratracheal instillation. Slides were scanned with Pathscan Enabler IV at 3600 dpi (Meyer Instruments, Houston, TX, USA) and are representative of four hamsters. Bar = 1 mm.

Our initial studies revealed that Ad14p1 induced a patchy bronchopneumonia at 7 days post hamster infection, whereas Ad14 induced a mild inflammatory response [18]. Preliminary data suggested that the observed difference in pathogenesis between Ad14 and Ad14p1 was not a result of delayed inflammatory response in Ad14-infected hamsters. To determine whether the divergent inflammatory responses were caused by viral factors rather than differences in the timing of the host responses to the two viruses, we infected Syrian hamsters with 5 × 10^9^ Ad14 (prototype deWit strain) or Ad14p1 viruses (as determined by qPCR) per hamster via intratracheal inoculation and sacrificed hamsters at the indicated time points up to 14 days post infection. Gross examination of hematoxylin and eosin (H&E) stained lungs showed that, at day 3 p.i., there were signs of inflammation in the lungs of animals infected with either Ad14 or Ad14p1, as evidenced by small, localized areas of increased cell density (Figure 1). By day 5 p.i. with Ad14p1, there was a marked increase in the infiltrating cell density in the larger left lung lobe with some localized infiltration to the medium-sized right lung lobe, but there was only minimal, localized inflammatory cell infiltration in the lung lobes of Ad14-infected hamsters. At day 7 p.i., there continued to be dense, patchy consolidation in all lung lobes of Ad14p1-infected animals, but the localized areas of inflammation in the lungs of Ad14-infected animals had begun to diminish. At day 10 p.i., the inflammatory infiltrate in the lungs of Ad14p1-infected animals had contracted into peribronchial areas, with persistent, multilobar involvement, whereas the lung infiltrates of Ad14-infected animals had mostly resolved. By day 14 p.i., the inflammatory infiltrates in the lungs of Ad14p1-infected animals had mostly resolved and left what appeared to be diffuse areas of decreased lung parenchyma. In contrast, at that time point, the lungs of animals infected with Ad14 had minimal evidence of inflammation and normal parenchymal architecture.

**Figure 2:**
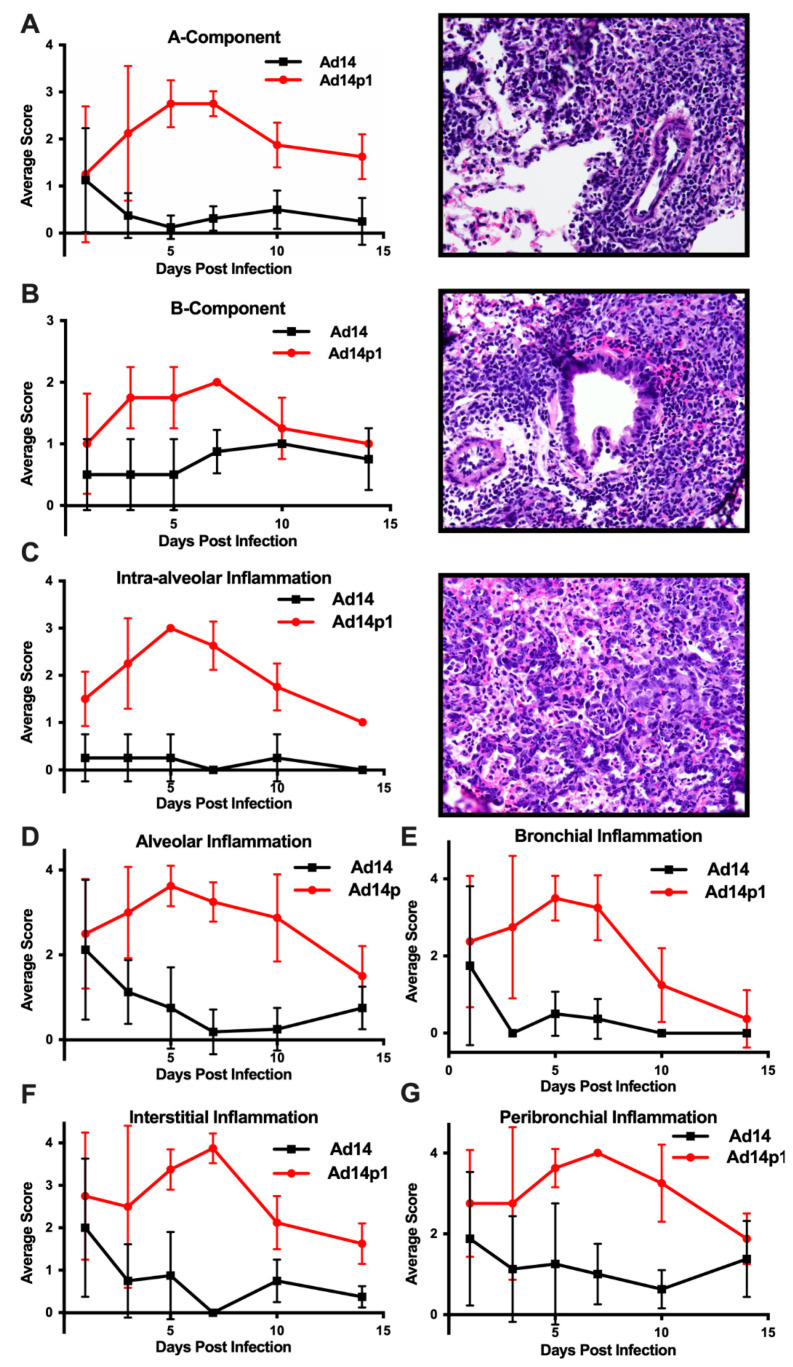
Intensity of Ad14 and Ad14p1 induced lung inflammation. H&E sections from Ad14- and Ad14p1-infected hamsters were scored by a pathologist for inflammation based on the lung allograft rejection scoring standards for (**A**) A-component and (**B**) B-component. H&E sections from Ad14- and Ad14p1-infected hamsters were scored from the same slides by a pathologist according the guidelines described in the methods section to determine regional pneumonitis: (**C**) Intra-alveolar (**D**) alveolar, (**E**) bronchial, (**F**) interstitial, and (**G**) peribronchial inflammatory scores. H&E sections from an Ad14p1-infected hamster at day 5 post infection. Representative 40× images of (**A**) Grade A3, A-component score, (**B**) high-grade B-component score, and (**C**) G3 intra-alveolar score. *n* = 4, Mean ± SD.

**Figure 3:**
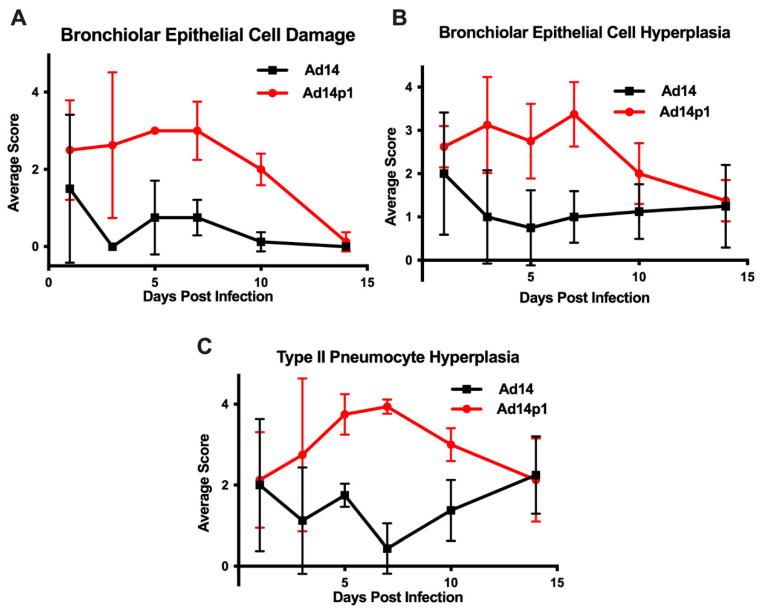
Effects of Ad14 and Ad14p1 on hamster lung cells. Airway epithelial cell changes were assessed by evaluating (**A**) bronchiolar epithelial cell damage, (**B**) bronchiolar epithelial cell hyperplasia, and (**C**) Type II pneumocyte hyperplasia. Scoring criteria are explained in the methods section. *n* = 4, Mean ± SD.

The intensity of lung inflammation was assessed by two board certified pathologists. Mononuclear infiltration of perivascular and interstitial spaces (A-component) and small airway inflammation (B-component) were scored according to the 2007 revision for grading lung allograft rejection [19]. Ad14p1 induced increasing mononuclear infiltration to moderate–severe levels at days 5 and 7 p.i. and regressed to mild levels at days 10 and 14 p.i. (Figure 2A, red line). In contrast, other than mild infiltration at day 1, Ad14 induced minimal to no mononuclear infiltrates in either the perivascular or interstitial spaces (Figure 2A, black line). Lymphocytic inflammation of the small airways (Figure 2B) increased during Ad14p1 infection (red line) from a low (day 1) to high (days 3–7) degree before decreasing to low degree at days 10–14, while Ad14 (black line) caused little inflammation until reaching low degree inflammation at days 7–14. Infection with Ad14p1 (red line) induced severe pneumonitis in alveolar, intra-alveolar, bronchial, peribronchial and interstitial areas that peaked at days 5 and 7 post infection and then began to resolve at days 10 and 14 (Figure 2C–G). Ad14p1 also induced bronchiolar epithelial cell damage (Figure 3A), bronchiolar epithelial cell hyperplasia (Figure 3B), and sloughing and necrosis of epithelial cells, none of which was observed in Ad14-infected hamsters. Infection with Ad14p1 also caused type II pneumocyte hyperplasia (Figure 3C, red line) that was not observed in Ad14-infected hamsters (Figure 3C, black line). Overall, these pathological scoring data supported and further defined the virus-specific differences in gross and microscopic lung inflammatory cell responses to comparable, initial infections with Ad14p1 or Ad14.

### 3.2. Ad14 and Ad14p1 Induce a Mixed Leukocyte Immune Response

**Figure 4:**
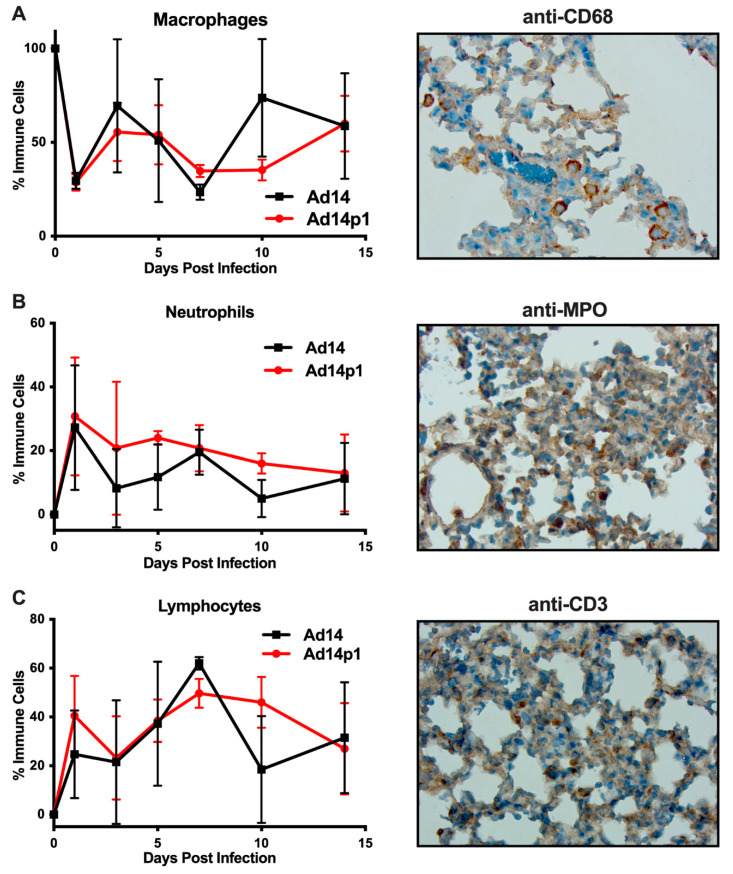
Inflammatory cell composition of Ad14- and Ad14p1-induced pneumonias. Differential cell counts for macrophages (**A**), neutrophils (**B**), and lymphocytes (**C**) were determined at the periphery of pneumonias by a blinded pathologist and expressed as the percentage of total inflammatory cells (mean ± SEM, *n* = 4). Immunohistochemistry was used to confirm the presence of macrophages with anti-CD63, neutrophils with anti-MPO, and lymphocytes with anti-CD3ε. Images (100×) are representative of four hamsters.

It was noted that the infiltrating inflammatory cells appeared to be primarily mononuclear cells. However, due to the thickness of the sections and the inflammatory cell density in the localized areas of pneumonia, it was difficult to determine differential counts of inflammatory cell types (Figure 2A, photo). Therefore, thinner sections were made and differential cell (macrophage, neutrophil, and lymphocyte) counts were done at the periphery of the inflamed zones. The inflamed zones in both Ad14- and Ad14p1-infected hamsters show a mixed leukocyte infiltration consisting of macrophages (Figure 4A), neutrophils (Figure 4B), and lymphocytes (Figure 4C). Immunohistochemistry was done to confirm the presence of macrophages using anti-CD68 (Figure 4A, photo), neutrophils using anti-MPO (Figure 4B, photo) and T-cells using anti-CD3ε (Figure 4C, photo). There was little difference in the timing of leukocyte infiltration in animals infected with the two viruses, but rather it was the intensity of leukocyte infiltration that was different between Ad14- and Ad14p1-infected hamsters.

**Figure 5:**
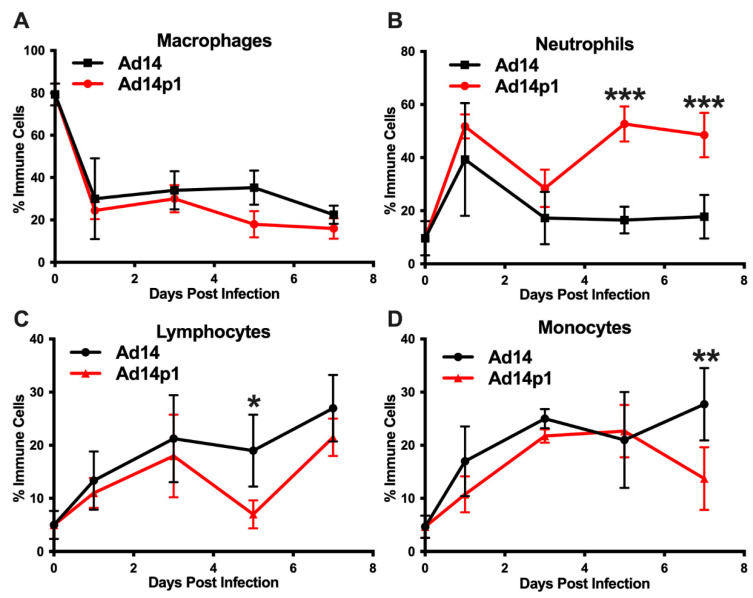
Effects of Ad14 and Ad14p1 on inflammatory cell composition of bronchoalveolar lavage (BAL) fluid. Syrian hamsters were infected with 5 × 10^9^ genomes/animal via intratracheal instillation. BAL was performed on the days indicated, and cytospins were performed to determine differential cell counts. Macrophages (**A**), neutrophils (**B**), lymphocytes (**C**), and monocytes (**D**) are expressed as the percentage of total inflammatory cells (mean ± SEM). A one-way ANOVA was performed with *p*-values determined by a post-hoc Sidak test (*n* = 4, *** *p* < 0.001, ** *p* < 0.01, * *p* < 0.05).

A second set of hamsters was infected with Ad14 or Ad14p1 to examine the immune cell infiltrates in bronchoalveolar lavage (BAL) fluids, as another measure of the lung inflammatory response. Infection with both Ad14 and Ad14p1 resulted in a similar decrease in the percentage of macrophages, compared with uninfected controls (Figure 5A), but showed a spike in BAL neutrophils at day 1 p.i. that then waned by day 3 p.i. (Figure 5B). The ratio of banded (immature) to segmented (mature) neutrophils was 1:4 for both Ad14- and Ad141p1-infected hamsters between days 1 and 3 post infection. Ad14p1-infected animals showed a second wave of infiltrating neutrophils in the BAL at day 5 p.i. that persisted at day 7, whereas there was no such second wave of neutrophil influx in Ad14-infected hamsters. This second neutrophil wave was associated with an increase in the ratio of banded to segmented neutrophils to 1:2 in Ad14p1-infected hamsters, indicating an influx of bone marrow derived, immature neutrophil forms at this point in infection. In addition, the BAL fluid collected from the Ad14p1-infected hamsters was significantly bloodier than that collected from Ad14-infected hamsters. Ad14- and Ad14p1-infected animals showed a similar influx of lymphocytes (Figure 5C) and monocytes (Figure 5D) over the course of infection.

### 3.3. Ad14 and Ad14p1 Replicate in Syrian Hamsters

**Figure 6:**
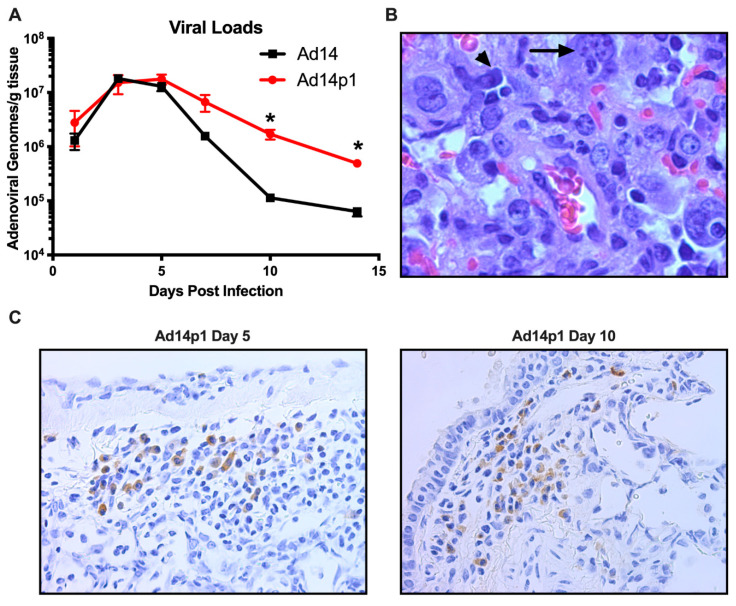
Permissive Ad14 and Ad14p1 infection in Syrian hamster lungs. (**A**) Viral loads were assessed at days 1, 3, 5, 7, 10, and 14 post infection by qPCR from homogenates of one of the right lung lobes and expressed as genomes/g tissue. Mean ± SEM, *n* = 4, one-way ANOVA followed by Holm–Sidak, * *p* < 0.05. (**B**) Presence of smudge cells (arrowhead) and inclusion bodes (arrow) in an H&E stained section of the lung of an Ad14p1-infected hamster at day 7 post infection (100×). (**C**) Immunohistochemistry staining of Ad14p1 with anti-hexon antibody (adenovirus 20/11 and 2/6; Cell Marque) in infected hamster lungs at day 5 and 10 post infection. Images are representative of four hamsters per time point.

Previously we have shown that, at day 7 p.i., there was little difference between the amount of Ad14 or Ad14p1 virus present in the lungs of infected hamsters [18]. In Figure 6A, we show that, during early time points in which the greatest inflammatory difference was observed (days 3–7 p.i.; Figure 2), there was no significant difference in the viral loads in the lungs of hamsters infected with Ad14 or Ad14p1. However, as the inflammation resolved at days 10 and 14 p.i. (Figure 2), there was a significantly greater persistence in the amount of Ad14p1 detected in infected hamster lungs, when compared with those of Ad14-infected animals. Animals infected with either virus showed histological signs of Ad infection, including smudge cells and nuclear inclusions (Figure 6B). In Figure 6C, IHC analysis, using an Ad-specific, anti-hexon antibody revealed that, at days 5 and 10 post infection, there were viral structural genes present in cells in the lungs of animals infected with Ad14p1. Overall inspection of lung sections from multiple animals indicated that there were more areas staining positive for Ad hexon in the day 5 lungs than in the lungs at day 10 post infection. Taken together, these data support the conclusion that Syrian hamsters are permissive for human adenovirus infection and replication and, therefore, are useful as a small animal model for studies of Ad14 and Ad14p1 pulmonary pathogenesis.

### 3.4. Ad14 and Ad14p1 Infections Are Associated with Different Cytokine/Chemokine Profiles

**Figure 7:**
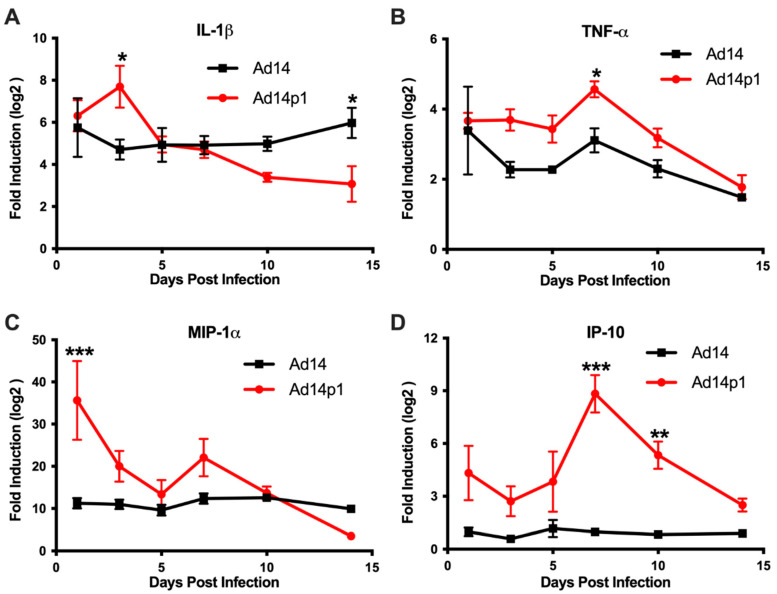
Cytokine expression in the lungs during Ad14 and Ad14p1 infection. (**A**) IL-1β, (**B**) TNF-α, (**C**) MIP-1α, and (**D**) IP-10 gene expression were determined by RT-qPCR of RNA from one of the right lung lobes of infected hamsters at the indicated times post infection. Fold change was determined by normalizing to RPL-18 expression and then by the 2 ΔΔCt method against uninfected control animals. Mean ± SEM, *n* = 4, one-way ANOVA followed by Holm–Sidak (*** *p* < 0.001, ** *p* < 0.01, * *p* < 0.05).

Based on the differences in the inflammatory cell responses observed after Ad14 and Ad14p1 infections, different patterns of expression of pro-inflammatory cytokines and chemokines would be predicted in the lungs of Ad14p1-infected hamsters compared to Ad14-infected hamsters. Cytokine/chemokine expression was assessed by qPCR from total RNA isolated from one of the right lobes from lungs of infected hamsters using primers previously validated for the Syrian hamster [20]. Infection of Syrian hamsters with either Ad14 or Ad14p1 induced expression of IL-1β (Figure 7A) and TNF-α (Figure 7B) at day 1 post infection. IL-1β levels remained constant during the 14 days following Ad14 infection; however, infection with Ad14p1 resulted in a further induction of IL-1β at day 3 p.i. that returned to a level comparable to that induced by Ad14 infection by day 5 post infection. Similarly, Ad14 infection induced a fairly constant increase in TNF-α expression during the 14 days p.i., whereas Ad14p1 infection induced higher TNF-α expression that peaked at day 7 p.i. before returning to levels comparable to those observed with Ad14 infection from days 10 to 14 p.i. The chemokine MIP-1α was also induced by infection with both Ad14 and Ad14p1 (Figure 7C). During Ad14 infection, induction of MIP-1α remained at the same level during 14 days p.i., whereas Ad14p1 infection induced greater expression of MIP-1α that spiked at day 1 p.i., with another slight, relative increase at day 7 p.i. before returning to levels similar to or lower than those observed with Ad14 infection at days 10 and 14 p.i. Figure 7D shows that there was little induction of IP-10 in the lungs of Ad14-infected hamsters compared to uninfected hamsters. In contrast, there were increased IP-10 levels in the lungs of Ad14p1-infected hamsters detected beginning at day 1 p.i. and peaking at days 7 and 10 p.i., before returning to the low levels observed with Ad14 infection by day 14 p.i. In summary, these data show that infection with both viruses induced cytokine and chemokine responses greater than the baseline levels detected in uninfected hamsters and, more importantly for this comparative immunopathological study, that there was a generally greater cytokine and chemokine response to infection with Ad14p1 than to infection with Ad14.

## 4. Discussion

Ad14p1 is an emergent strain of Ad14 that has induced outbreaks of respiratory disease in both military and civilian populations and can induce severe respiratory disease that can result in ARDS. The mechanisms through which Ad14p1 induces a more severe acute lung injury than Ad14 or other Ad14 variants is unknown and has been postulated to be host dependent [5,6,7,8,11,13,14,21,22,23]. We have reported that the reduced cellular expression of the adenoviral *E1B* 20K gene that is observed during Ad14p1 infection results in abrogation of the anti-inflammatory effects of infected cells that is normally observed with cells infected with wild type Ad14 [18]. As an apparent result of this loss-of-function viral phenotype, there is a net enhancement in the host inflammatory response to Ad14p1 infection, for example as evidenced by increased bronchopneumonia following respiratory infection with Ad14p1 vs. Ad14. The objective of the current study was to characterize this hamster model of adenoviral infection by studying the comparative intensity and kinetics of the host immune response over two weeks following infection with Ad14p1 vs. Ad14, to further define the utility of the Syrian hamster model for analysis of adenovirus-induced acute lung injury as the early phase of the host ARDS-like response to Ad14p1 infection.

The data show that the comparative reduction in the lung inflammatory response to Ad14 vs. Ad14p1 infection is not caused by a slower initial host response to infection (Figure 1 and Figure 2) or reduced viral replication (Figure 6), but rather is due to a virus-specific effect that results in a more intense innate immune response to Ad14p1 infection. Both Ad14 and Ad14p1 induced a mixed leukocyte infiltration into the lung and bronchoalveolar spaces (Figure 4 and Figure 5), but there was a quantitative increase in selected chemokines and cytokines and the associated leukocyte infiltration during Ad14p1 infection. This was specifically evidenced by the second wave of neutrophil infiltration (Figure 5B) and increased lung tissue cytokine and chemokine (Figure 7) expression observed in Ad14p1, but not Ad14, infected hamsters. Based upon our previous viral genetic and inflammatory response studies, we postulate that this enhanced lung immunopathogenesis of Ad14p1 is mediated by the reduced expression of the *E1B* 20K gene [17,18]. Work is underway to further test this hypothesis and define the cellular mechanisms through which reduced *E1B* 20K gene expression causes increased host inflammatory responses to viral infection.

These data and other studies indicate that the Syrian hamster is a good small animal model for studies on adenovirus induced lung immunopathogenesis. There are limited studies of Ad respiratory infection in the Syrian hamster with HAdV-C5 (Ad5) and HAdV-C6 (Ad6). Most of the hamster studies have focused on systemic infection via i.v. injection. Similar to our results, respiratory infection with Ad6 resulted in a mixed leukocyte infiltration and cytokine expression in the lung [24]. Those studies revealed that intranasal lung infection with Ad5 or Ad6 resulted in various levels of viral replication, depending on the viral inoculum. In studies where the infectious dose was similar to that used in our studies, similar levels of viral replication were observed [24,25,26]. Prince and colleagues used the cotton rat to study Ad5 induced pneumonia [27]. Like Syrian hamsters, cotton rats are permissive for Ad5 replication, and Ad lung infection of these animals results in pneumonia. The data presented here show that Ad14 infection causes a productive infection in Syrian hamsters resulting in a limited peribronchial infection but that infection with the emergent Ad14p1 strain of Ad14 induces a more intense, patchy bronchopneumonia that can be associated with lung parenchymal loss as a possible consequence of the initial, acute lung injury (Figure 1). Infection of Syrian hamsters with either Ad14 or Ad14p1 resulted in infiltration of neutrophils, lymphocytes, and macrophages (Figure 4). Yei, et al. showed that infection of cotton rats is associated with a mixed leukocyte response early during infection that evolves to a lymphocyte predominant response at late times after infection, which is consistent with our findings in hamsters [28]. The pathology findings presented here are consistent with the findings in the human case of Ad14p1 infection noted in the Introduction, suggesting that the Syrian hamster is a relevant model system to study Ad14 and Ad14p1 pathogenesis [14].

The increased expression of pro-inflammatory cytokines and chemokines during Ad14 and Ad14p1 infection is similar to that observed in cotton rats, as well as in non-permissive adenoviral infection of mice [29,30,31]. This cytokine/chemokine response can be seen after challenges with inactivated virus or viral infection of non-permissive animals suggesting that early cytokine/chemokine responses might be triggered by virion interactions with cellular receptors, early viral gene expression or by both of these early steps in viral infection, without completion of the viral replication cycle. The details of this proposed virus interaction with host cell receptors remain to be defined.

The scavenger receptor, MARCO, is expressed on alveolar macrophages and has been shown to be a receptor for multiple adenovirus serotypes [32,33]. Interaction of MARCO and Ad5 can stimulate macrophage production of IL-6, IL-1β and IFN α/β; therefore, it is possible that the MARCO:Ad14/Ad14p1 interaction at day 1 p.i. was involved in the increased expression of the cytokines and chemokines observed in our studies (Figure 7) [32]. It is also possible that the MARCO interaction allows viral binding to other cell surface receptors like TLR2 and TLR4, through which Ad can induce expression of MIP-1α [34,35]. Interaction with MARCO has been mapped to the adenovirus hypervariable region 1 of Ad hexon. How Ad interact with TLRs is unknown [33]. Ad interacts with integrins and the coxsackievirus and adenovirus receptor (CAR) for group C Ad and desmoglein-2 (DSG-2) for group B Ad including Ad14/Ad14p1. However, neither CAR nor DSG-2 are expressed on AM suggesting a key role for MARCO in Ad interactions with AM [36,37]. Understanding the role of MARCO expression on AM in regulating Ad14p1 pathogenesis is currently under investigation.

Early during hamster infection, the lung viral loads were identical for Ad14 and Ad14p1; however, at days 10 and 14 p.i., the viral loads for Ad14p1 were higher than for Ad14 (Figure 6A). The observations that Ad14p1 infection induced a more intense inflammatory response by day 5 p.i. (Figure 1 and Figure 2) but the viral load in lung tissue cleared more slowly than for Ad14 thereafter would appear to be contradictory, if there were a simple, direct correlation between increased inflammation and decreased viral titer. Therefore, these results suggest that there is a more complex correlation between viral clearance and the host inflammatory response. It is unlikely that these differences are explained by differences in viral replication rates, since the two viral titers were identical in the lungs through the first five days of infection and since Ad14 and Ad14p1 replicate with equal efficiency in mammalian cells [15]. It is possible that the bronchial cell and type II cell hyperplasia observed with Ad14p1 infection might provide an increased number of host cells for ongoing viral infection and replication that could contribute to delayed Ad14p1 clearance. A similar host cell infection effect on viral load could have involved infiltrating lymphocytes, since adenovirus can replicate in both T- and B-lymphocytes [38]. Virus-specific differences in effects on macrophage function could also be a factor. For example, MARCO expression on alveolar macrophages decreases during influenza infection [39]. If a similar reduction in macrophage scavenger receptor expression or function occurred during Ad14p1 infection but to a lesser extent during Ad14 infection, then Ad14p1 sequestration might be reduced, compared to Ad14. Whether uptake of Ad by alveolar macrophages results in cell death of the macrophages is unclear, but, if alveolar macrophages die as a result of infection, the decreased numbers of alveolar macrophages could also partly account for the increased viral load of Ad14p1 at days 10 and 14 post infection. As a summary generalization, we conclude that the net effects of the differential cytokine, chemokine and innate immune cell antiviral activities contributed to the relative difference in the rates of viral load reduction during the second week post infection.

The clinical presentation of ARDS is based on the Berlin definition, which states that patients display bilateral pulmonary infiltrates, hypoxemia (PaO_2_/FiO_2_ ratio < 300 mmHg) and lung edema that cannot be explained by cardiac failure or fluid overload [40]. The American Thoracic Society (ATS) workshop report established a set of four criteria for ARDS in animal models of which three must be met. Based on the assumption that the clinical definition was not practical for small animal models, there should be: histological evidence of tissue injury; alterations of the alveolo–capillary barrier; an inflammatory response; and evidence of physiological dysfunction, the preferred parameter being hypoxemia [41]. Our data demonstrate that Ad14p1 infection results in bilateral pulmonary infiltrates (Figure 1) and alterations in the alveolo–capillary barrier (Figure 4 and Figure 5). Diffuse alveolar damage was observed in Ad14p1-infected lungs including bronchiolar epithelial cell damage and hyperplasia (Figure 3A,B), interstitial acute inflammation (Figure 2F), and type II pneumocyte hyperplasia (Figure 3C); however, little hyaline membrane formation was observed, which is not uncommon in animals compared to humans [42]. We observed a strong inflammatory response including neutrophil and lymphocyte infiltration (Figure 4 and Figure 5), as well as increased cytokine/chemokine expression in the lungs (Figure 7). Our Syrian hamster model of Ad14p1 infection meets the ATS criteria for ARDS; however, without evidence of hypoxemia, we are not comfortable with describing the outcome as ARDS. Most current small animal models die of shock rather than ARDS within 72 h of injury and therefore do not allow for multiple-hit events that trigger ARDS as is seen in humans [43]. We propose that this Syrian hamster model of Ad14p1-induced acute lung injury can be used as a small animal model system with which to study the early events during viral infection that can trigger lung immunopathology that might evolve to an ARDS-like lung injury. The unique feature of this virus infection model is the availability of a comparative model in which altered expression of a single viral gene (*E1B* 20K) might control the differential inflammatory response that determines the course of lung injury following infection that can evolve to either minimal inflammation and rapid resolution (E1B 20K-high (wild type Ad14) state) or intense bronchopneumonia and inflammation-induced lung damage (E1B 20K-low (Ad14p1) state). The hypothesis that is the basis of this comparative viral pathogenesis model is that reduced *E1B* 20K expression during Ad14p1 infection results in infected cells that lack the Ad14 phenotype of immunorepressive activity for innate immune effector cells, which results in a marked increase in virally triggered cytokine/chemokine-mediated innate immune response activation and associated lung immunopathology [18]. This model will be useful for translational studies to test that hypothesis, as well as for small animal studies of other emerging Ad variants that are associated with increased lung immunopathogenesis.

## References

[1] Van der Veen J., Kok G. (1957). Isolation and typing of adenoviruses recovered from military recruits with acute respiratory disease in The Netherlands. Am. J. Epidemiol..

[2] Metzgar D., Osuna M., Kajon A.E., Hawksworth A.W., Irvine M., Russell K.L. (2007). Abrupt emergence of diverse species B adenoviruses at US military recruit training centers. J. Infect. Dis..

[3] Houng H.S.H., Gong H., Kajon A.E., Jones M.S., Kuschner R.A., Lyons A., Lott L., Lin K.-H., Metzgar D. (2010). Genome sequences of human Adenovirus 14 isolates from mild respiratory cases and a fatal pneumonia, isolated during 2006-2007 epidemics in North America. Respir. Res..

[4] Zhang Q., Jing S., Cheng Z., Yu Z., Dehghan S., Shamsaddini A., Yan Y., Li M., Seto D. (2017). Comparative genomic analysis of two emergent human adenovirus type 14 respiratory pathogen isolates in China reveals similar yet divergent genomes. Emerg. Microbes Infect..

[5] Mi Z., Butt A.M., An X., Jiang T., Liu W., Qin C., Cao W.C., Tong Y. (2013). Genomic analysis of HAdV-B14 isolate from the outbreak of febrile respiratory infection in China. Genomics.

[6] Tang L., An J., Xie Z., Dehghan S., Seto D., Xu W., Ji Y. (2013). Genome and bioinformatic analysis of a HAdV-B14p1 virus isolated from a baby with pneumonia in Beijing, China. PLoS ONE.

[7] Carr M.J., Kajon A.E., Lu X., Dunford L., O’Reilly P., Holder P., De Gascun C.F., Coughlan S., Connell J., Erdman D.D. (2011). Deaths associated with human adenovirus-14p1 infections, Europe, 2009–2010. Emerg. Infect. Dis..

[8] Lewis P.F., Schmidt M.A., Lu X., Erdman D.D., Campbell M., Thomas A., Cieslak P.R., Grenz L.D., Tsaknardis L., Gleaves C. (2009). A community-based outbreak of severe respiratory illness caused by human adenovirus serotype 14. J. Infect. Dis..

[9] Louie J.K., Kajon A.E., Holodniy M., Guardia-LaBar L., Lee B., Petru A.M., Hacker J.K., Schnurr D.P. (2008). Severe pneumonia due to adenovirus serotype 14: A new respiratory threat?. Clin. Infect. Dis. Off. Publ. Infect. Dis. Soc. Am..

[10] Tate J.E., Bunning M.L., Lott L., Lu X., Su J., Metzgar D., Brosch L., Panozzo C.A., Marconi V.C., Faix D.J. (2009). Outbreak of severe respiratory disease associated with emergent human adenovirus serotype 14 at a US air force training facility in 2007. J. Infect. Dis..

[11] O’Flanagan D., O’Donnell J., Domegan L., Fitzpatrick F., Connell J., Coughlan S., De Gascun C., Carr M.J. (2011). First reported cases of human adenovirus serotype 14p1 infection, Ireland, October 2009 to July 2010. Euro. Surveill..

[12] Esposito D.H., Gardner T.J., Schneider E., Stockman L.J., Tate J.E., Panozzo C.A., Robbins C.L., Jenkerson S.A., Thomas L., Watson C.M. (2010). Outbreak of pneumonia associated with emergent human adenovirus serotype 14--Southeast Alaska, 2008. J. Infect. Dis..

[13] Kajon A.E., Lu X., Erdman D.D., Louie J., Schnurr D., George K.S., Koopmans M.P., Allibhai T., Metzgar D. (2010). Molecular epidemiology and brief history of emerging adenovirus 14-associated respiratory disease in the United States. J. Infect. Dis..

[14] Girouard G., Garceau R., Thibault L., Oussedik Y., Bastien N., Li Y. (2013). Adenovirus serotype 14 infection, New Brunswick, Canada, 2011. Emerg. Infect. Dis..

[15] Anderson B.D., Barr K.L., Heil G.L., Friary J.A., Gray G.C. (2012). A comparison of viral fitness and virulence between emergent adenovirus 14p1 and prototype adenovirus 14p strains. J. Clin. Virol..

[16] Lam E., Ramke M., Warnecke G., Schrepfer S., Kopfnagel V., Dobner T., Heim A. (2015). Effective Apical Infection of Differentiated Human Bronchial Epithelial Cells and Induction of Proinflammatory Chemokines by the Highly Pneumotropic Human Adenovirus Type 14p1. PLoS ONE.

[17] Radke J.R., Grigera F., Ucker D.S., Cook J.L. (2014). Adenovirus E1B 19-kilodalton protein modulates innate immunity through apoptotic mimicry. J. Virol..

[18] Radke J.R., Yong S.L., Cook J.L. (2016). Low-Level Expression of the E1B 20-Kilodalton Protein by Adenovirus 14p1 Enhances Viral Immunopathogenesis. J. Virol..

[19] Stewart S., Fishbein M.C., Snell G.I., Berry G.J., Boehler A., Burke M.M., Glanville A., Gould F.K., Magro C., Marboe C.C. (2007). Revision of the 1996 working formulation for the standardization of nomenclature in the diagnosis of lung rejection. J. Heart Lung Transplant..

[20] Zivcec M., Safronetz D., Haddock E., Feldmann H., Ebihara H. (2011). Validation of assays to monitor immune responses in the Syrian golden hamster (Mesocricetus auratus). J. Immunol. Methods.

[21] Zhang Q., Seto D., Zhao S., Zhu L., Zhao W., Wan C. (2012). Genome Sequence of the First Human Adenovirus Type 14 Isolated in China. J. Virol..

[22] Parcell B.J., McIntyre P.G., Yirrell D.L., Fraser A., Quinn M., Templeton K., Christie S., Romanes F. (2015). Prison and community outbreak of severe respiratory infection due to adenovirus type 14p1 in Tayside, UK. J. Public Health (Oxf.).

[23] Huang G., Yu D., Zhu Z., Zhao H., Wang P., Gray G.C., Meng L., Xu W. (2013). Outbreak of febrile respiratory illness associated with human adenovirus type 14p1 in Gansu Province, China. Influenza Other Respi. Viruses.

[24] Ying B., Toth K., Spencer J.F., Aurora R., Wold W.S.M. (2015). Transcriptome sequencing and development of an expression microarray platform for liver infection in adenovirus type 5-infected Syrian golden hamsters. Virology.

[25] Thomas M.A., Spencer J.F., La Regina M.C., Dhar D., Tollefson A.E., Toth K., Wold W.S.M. (2006). Syrian hamster as a permissive immunocompetent animal model for the study of oncolytic adenovirus vectors. Cancer Res..

[26] Tollefson A.E., Ying B., Spencer J.F., Sagartz J.E., Wold W.S.M., Toth K. (2017). Pathology in Permissive Syrian Hamsters after Infection with Species C Human Adenovirus (HAdV-C) Is the Result of Virus Replication: HAdV-C6 Replicates More and Causes More Pathology than HAdV-C5. J. Virol..

[27] Prince G.A., Porter D.D., Jenson A.B., Horswood R.L., Chanock R.M., Ginsberg H.S. (1993). Pathogenesis of adenovirus type 5 pneumonia in cotton rats (Sigmodon hispidus). J. Virol..

[28] Yei S., Mittereder N., Wert S., Whitsett J.A., Wilmott R.W., Trapnell B.C. (1994). In vivo evaluation of the safety of adenovirus-mediated transfer of the human cystic fibrosis transmembrane conductance regulator cDNA to the lung. Hum. Gene Ther..

[29] Ginsberg H.S., Moldawer L.L., Prince G.A. (1999). Role of the type 5 adenovirus gene encoding the early region 1B 55-kDa protein in pulmonary pathogenesis. Proc. Natl. Acad. Sci. USA.

[30] McCoy R.D., Davidson B.L., Roessler B.J., Huffnagle G.B., Janich S.L., Laing T.J., Simon R.H. (1995). Pulmonary inflammation induced by incomplete or inactivated adenoviral particles. Hum. Gene Ther..

[31] Otake K., Ennist D.L., Harrod K., Trapnell B.C. (1998). Nonspecific inflammation inhibits adenovirus-mediated pulmonary gene transfer and expression independent of specific acquired immune responses. Hum. Gene Ther..

[32] Maler M.D., Nielsen P.J., Stichling N., Cohen I., Ruzsics Z., Wood C., Engelhard P., Suomalainen M., Gyory I., Huber M. (2017). Key Role of the Scavenger Receptor MARCO in Mediating Adenovirus Infection and Subsequent Innate Responses of Macrophages. MBio.

[33] Stichling N., Suomalainen M., Flatt J.W., Schmid M., Pacesa M., Hemmi S., Jungraithmayr W., Maler M.D., Freudenberg M.A., Plückthun A. (2018). Lung macrophage scavenger receptor SR-A6 (MARCO) is an adenovirus type-specific virus entry receptor. PLoS Pathog..

[34] Appledorn D.M., Patial S., McBride A., Godbehere S., Van Rooijen N., Parameswaran N., Amalfitano A. (2008). Adenovirus vector-induced innate inflammatory mediators, MAPK signaling, as well as adaptive immune responses are dependent upon both TLR2 and TLR9 in vivo. J. Immunol..

[35] Zhou X., Ramke M., Chintakuntlawar A.V., Lee J.Y., Rajaiya J., Chodosh J. (2017). Role of MyD88 in adenovirus keratitis. Immunol. Cell Biol..

[36] Wang H., Li Z.-Y., Liu Y., Persson J., Beyer I., Möller T., Koyuncu D., Drescher M.R., Strauss R., Zhang X.-B. (2010). Desmoglein 2 is a receptor for adenovirus serotypes 3, 7, 11 and 14. Nat. Med..

[37] Wang H., Tuve S., Erdman D.D., Lieber A. (2009). Receptor usage of a newly emergent adenovirus type 14. Virology.

[38] Lavery D., Fu S.M., Lufkin T., Chen-Kiang S. (1987). Productive infection of cultured human lymphoid cells by adenovirus. J. Virol..

[39] Wu M., Gibbons J.G., DeLoid G.M., Bedugnis A.S., Thimmulappa R.K., Biswal S., Kobzik L. (2017). Immunomodulators targeting MARCO expression improve resistance to postinfluenza bacterial pneumonia. Am. J. Physiol. Lung Cell Mol. Physiol..

[40] Ranieri V.M., Rubenfeld G.D., Thompson B.T., Ferguson N.D., Caldwell E., Fan E., Camporota L., Slutsky A.S., ARDS Definition Task Force (2012). Acute respiratory distress syndrome: The Berlin Definition. J. Am. Med. Assoc..

[41] Matute-Bello G., Downey G., Moore B.B., Groshong S.D., Matthay M.A., Slutsky A.S., Kuebler W.M. (2011). Acute Lung Injury in Animals Study Group An official American Thoracic Society workshop report: Features and measurements of experimental acute lung injury in animals. Am. J. Respir. Cell Mol. Biol..

[42] Aeffner F., Bolon B., Davis I.C. (2015). Mouse Models of Acute Respiratory Distress Syndrome: A Review of Analytical Approaches, Pathologic Features, and Common Measurements. Toxicol. Pathol..

[43] Uhlig S., Kuebler W.M. (2018). Difficulties in modelling ARDS (2017 Grover Conference Series). Pulm. Circ..

